# Binding of α_v_β_3_ Integrin-Specific Radiotracers Is Modulated by Both Integrin Expression Level and Activation Status

**DOI:** 10.1007/s11307-017-1100-z

**Published:** 2017-07-10

**Authors:** Alexandra Andriu, Julie Crockett, Sergio Dall’Angelo, Monica Piras, Matteo Zanda, Ian N. Fleming

**Affiliations:** 1 Aberdeen Biomedical Imaging Centre, Institute of Medical Sciences, University of Aberdeen, Aberdeen, AB25 2ZD UK; 2 Arthritis and Musculoskeletal Medicine Research Programme, Division of Applied Medicine, Institute of Medical Sciences, University of Aberdeen, Aberdeen, AB25 2ZD UK; 3 Kosterlitz Centre for Therapeutics, Institute of Medical Sciences, University of Aberdeen, Aberdeen, Scotland AB25 2ZD UK

**Keywords:** Tumour angiogenesis, α_v_β_3_ integrin, Integrin signalling, Response assessment, PET imaging, Radiolabelled RGD peptides

## Abstract

**Purpose:**

Molecular imaging of α_v_β_3_ integrin has exhibited real potential to guide the appropriate use of anti-angiogenic therapies. However, an incomplete understanding of the factors that influence binding of α_v_β_3_ integrin-specific radiotracers currently limits their use for assessing response to therapy in cancer patients. This study identifies two fundamental factors that modulate uptake of these radiotracers.

Procedures

Experiments were performed in prostate cancer (PC3) and glioblastoma (U87MG) cells, which differentially express α_v_β_3_ integrin. α_v_β_3_ integrin-specific radiotracers were used to investigate the effect of manipulating α_v_β_3_ integrin expression or activation in cellular binding assays. β_3_ integrin and α_v_β_3_ integrin expression were measured by western blotting and flow cytometry, respectively. The effect of select pharmacological inhibitors on α_v_β_3_ integrin activation and expression was also determined.

**Results:**

Radiotracer binding was proportional to α_v_β_3_ integrin expression when it was decreased (β_3_ knock-down cells) or increased, either using pharmacological inhibitors of cell signalling or by culturing cells for different times. Studies with both small molecule and arginine–glycine–aspartic acid (RGD)-based radiotracers revealed increased radiotracer binding after activation of α_v_β_3_ integrin with Mn^2+^ or talin head domain. Moreover, inhibition of fundamental signalling pathways (mitogen-activated protein kinase kinase (MEK), Src and VEGFR2) decreased radiotracer binding, reflecting reduced α_v_β_3_ integrin activity.

**Conclusion:**

Binding of small molecule ligands and radiolabelled RGD peptides is modulated by expression and activation status of α_v_β_3_ integrin. α_v_β_3_ integrin-specific radiotracers can provide otherwise inaccessible information of the effect of signalling pathways on α_v_β_3_ integrin. This has significant implications for assessing response to anti-angiogenic therapies in clinical studies.

**Electronic supplementary material:**

The online version of this article (doi:10.1007/s11307-017-1100-z) contains supplementary material, which is available to authorized users.

## Introduction

Tumour angiogenesis, the formation of new blood vessels, is a hallmark of cancers that allows them to grow beyond a critical size (2–3 mm) and metastatize to other organs [1]. The angiogenic switch is triggered by tumour growth which creates a hypoxic and acidic environment, promoting release of pro-angiogenic factors such as vascular endothelial growth factor (VEGF) and development of new blood vessels and endothelial cell migration *via* integrins [1]. Therapeutic interventions that target VEGF receptor 2 (VEGFR2) and integrins have been evaluated as anti-angiogenic treatments, in accordance with their key roles in the pathogenesis of tumour angiogenesis [2, 3]. However, effective imaging methods are needed to assess whether tumours are actually responding to therapy, as the efficacy of these treatments varies considerably between tumour types and individual cancer patients.

The integrin family comprises 24 transmembrane receptors formed by heterodimeric combinations of 18 α and 8 β subunits. Each subunit comprises a short cytoplasmic domain, a single transmembrane region and an extracellular domain. Ligand binding to the extracellular domain allows integrins to collate information about the extracellular environment [4, 5]. In addition, their cytoplasmic domains recruit intracellular proteins such as talin, focal adhesion kinase (FAK) and Src, leading to activation of canonical signalling pathways. As a result of these interactions, integrins change their conformation (*i.e.,* undergo activation or inactivation) thereby driving tumour angiogenesis [6, 7].

Molecular imaging of α_v_β_3_ integrin offers a specific and quantitative method of assessing the angiogenic potential of tumours [8]. α_v_β_3_ integrin is highly expressed on angiogenic endothelial cells, involved in cell adhesion [9], cell migration and metastasis [2] and is a validated target for assessing tumour angiogenesis [10]. Vitronectin and fibronectin bind selectively to this receptor through an arginine–glycine–aspartic acid (RGD) recognition sequence. Multiple positron emission tomography (PET) radiotracers have been designed based on the RGD motif to provide information on tumour vasculature, with [^18^F]Galacto-RGD [11] and [^18^F]Fluciclatide [12] being the best characterised.

Clinical studies [11–13] and mouse xenograft experiments [14, 15] have both observed correlation between α_v_β_3_ integrin radiotracer uptake and baseline α_v_β_3_ integrin expression, supporting the use of these radiotracers as surrogate markers of tumour angiogenic potential. Clinical studies have not yet endorsed these radiotracers for assessing response to therapy, despite their considerable potential in this role [16]. One key reason is our incomplete understanding of how molecular mechanisms influence radiotracer uptake; two preclinical studies that have compared radiotracer binding with α_v_β_3_ integrin expression after anti-angiogenic therapy observed changes in radiotracer binding that could not be attributed to altered α_v_β_3_ expression [17, 18]. These reports strongly suggest that there are uncharacterised factor(s) that can influence binding of these radiotracers to cells/tumours.

In this study, we present conclusive evidence that binding of α_v_β_3_ integrin radiotracers to cells is influenced by both the expression level and activation status of the target receptor. Moreover, we also demonstrate that inhibition of fundamental signalling pathways (mitogen-activated protein kinase kinase (MEK), Src and VEGFR2) influences α_v_β_3_ integrin radiotracer binding, resulting from a change in integrin expression or reflecting decreased binding affinity. These results broaden our understanding of the molecular changes caused by anti-angiogenic treatment and have significant implications for the use of α_v_β_3_ integrin-specific radiotracers in assessing response to therapy with these agents.

## Materials and Methods

### Reagents

All reagents were purchased from Sigma-Aldrich, unless stated otherwise. UO126, PP-2, PF573228 and ZM323881 were from Bio-Techne. JetPRIME® transfection reagent was from Source BioScience. Talin head domain (THD) construct [19] was a kind gift from Prof David A. Calderwood (Yale University, USA). Anti-α_v_β_3_ integrin MAB1976 (LM609) was from Merck Millipore.

### Cell Lines and Culture Conditions

All cell lines used in this study (prostate cancer (PC)3 and U87MG) were authentic and purchased from ATCC. These cell lines were specifically selected because they are known to express moderate and high levels of α_v_β_3_, respectively. Cells were cultured in RPMI medium supplemented with 10 % FCS and 100 units/ml penicillin and 100 μg/ml streptomycin under sterile conditions at 37 °C in a humidified atmosphere containing 5 % CO_2_.

### Western Blotting

PC3 and U87MG cells were seeded at 0.35 × 10^6^ cells/60 mm plate. On the next day, cells were incubated with the specified concentrations of UO126, ZM323881, PF573228 or PP2 for the time indicated in each experiment. Cells were then washed with PBS and scraped into lysis buffer (PBS containing 1 mM sodium orthovanadate, 1 mM sodium pyrophosphate, dithiothreitol and protease inhibitors) and lysed by sonication. The total cell lysate was used to ensure that all blots are fully representative of the total cellular expression of each protein. The protein concentration in each sample was determined using the bicinchoninic acid (BCA) assay. Western blotting was performed essentially using the method indicated previously [20] with minor modifications using primary antibodies against the following proteins: β_3_ integrin, Santa Cruz Biotechnology; extracellular signal regulated kinase (ERK)1 and 2, pThr^202^/Tyr^204^ ERK 1 and 2, VEGFR2, pTyr^1175^ VEGFR2, FAK, pTyr^397^ FAK, Src and pTyr^416^ Src all from Cell Signalling; and green fluorescent protein (GFP) MAB3580 from EMD Merck Millipore. After incubation with primary antibody, membranes were washed in PBS containing 0.1 % Tween 20, then incubated for 1 h with the appropriate IR dye-conjugated secondary antibody (LI-COR) diluted 1:10,000. Images were captured with an Odyssey® CLx LI-COR imaging system. The densitometry of each protein band was measured using Image Studio Lite.

### Flow Cytometry Analysis of α_v_β_3_ Integrin Expression

PC3 and U87MG cells were seeded at 0.35 × 10^6^ cells/60 mm plate and grown for 72 h before the experiment, unless stated otherwise. Cells were harvested from plates using cell dissociation reagent. α_v_β_3_ cell surface expression was determined by flow cytometry as previously described [21], using the LM609 antibody which binds selectively to α_v_β_3_ integrin and detected with an Alexa Fluor 488 secondary antibody. Data were normalised against the untreated control, unless otherwise stated.

### *In Vitro* Radiotracer Binding Assay

ZMPZAT71 is a novel triazole-based α_v_β_3_ selective radiotracer with high α_v_β_3_ binding affinity designed at the University of Aberdeen [22]. Experiments were either performed with [^3^H]ZMPZAT71 (synthesised by RC TRITEC Ltd., Switzerland) or with [^18^F]FDR-Aoa-c(RGDfK) [21]. Cells were seeded at 0.35 × 10^6^ cells/60 mm plate and grown for 72 h, unless stated otherwise. On the day of the experiment, medium in each plate was replaced with 3 ml fresh RPMI containing 0.1 % BSA. [^3^H]ZMPZAT71 was added at 0.5 MBq/plate and incubated for 30 min (maximum radiotracer binding occurred within 30 min; data not shown). Unbound radioactivity was removed by rinsing the plates five times with ice-cold PBS. Cells were detached using 0.35 ml trypsin and neutralised with 0.35 ml RPMI. The radioactivity present in 0.5 ml cell suspension was measured in a TRICARB 2100 TR scintillation counter using ultima gold scintillation fluid. The protein content in the remaining 0.2 ml was measured using the BCA assay. Binding was expressed as a function of radioactivity added per plate and normalised to protein content. Radiotracer binding was performed in the presence and absence of 10 μM Cyclo(Arg-Gly-Asp-D-Phe-Lys) peptide (cRGDfK) (a non-radiolabelled cyclic peptide known to bind selectively to α_v_β_3_ integrin) [21]. Binding in the absence of cRGDfK represents total binding. Binding in presence of cRGDfK signifies non-specific binding. The difference between total and non-specific binding denotes specific binding (Fig. S1).

### β_3_ Integrin Knock-Down Clones

U87MG cells with decreased α_v_β_3_ integrin expression were generated by transfecting cells with a small hairpin RNA (shRNA) vector that specifically targets the integrin β_3_ subunit (GeneCopoeia, Rockville, USA). Cells that stably incorporated the shRNA vector were selected using puromycin. Individual cells were selected and allowed to proliferate prior to analysis for β_3_ knock-down by western blot and flow cytometry.

### Talin Head Domain Transfection

U87MG and PC3 cells were seeded at 0.35 × 10^6^ cells per 60 mm plate and incubated overnight. Cells were then transfected with either a THD construct linked to GFP or mock transfected, using JetPRIME reagent according to manufacturer’s instructions. Transfection efficiency in both cell lines was optimised by measuring the number of green cells at various times (4–48 h) after THD-GFP transfection using an EVOS AMG fluorescence digital inverted microscope.

### Statistical Analysis

Microsoft Office Excel 2013 for Windows and GraphPad Prism 5 were used for data processing, graph plotting and statistical analysis. Statistical analysis of all data was performed using two-tailed Student’s *t* test for two groups or one-way analysis of variance (ANOVA) for three or more groups. *P* values less than 0.05 were considered statistically significant.

## Results

### Radiotracer Binding Reflects α_v_β_3_ Expression Level

**Fig. 1.**
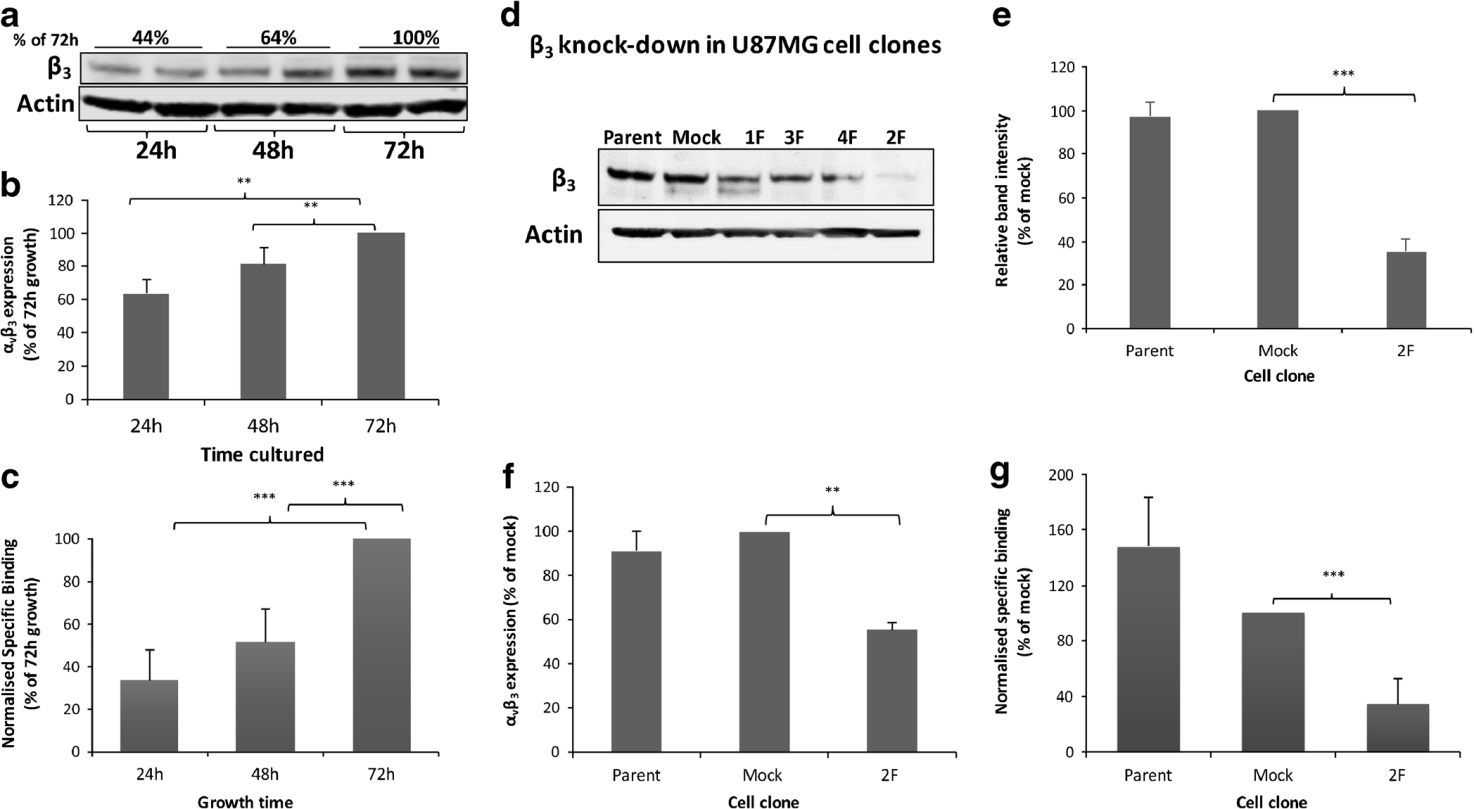
α_v_β_3_ integrin radiotracer binding reflects α_v_β_3_ integrin expression. U87MG cells were grown for 24, 48 or 72 h prior to analysis (**a**–**c**). **a** Representative β_3_ integrin western blot with band quantification values stated. **b** Cell surface α_v_β_3_ expression by flow cytometry. **c** [^3^H]ZMPZAT71 radiotracer binding assay. **d**–**g** Analysis of U87MG cells with different β_3_ integrin levels (untransfected parent, mock transfected and 2F β_3_ integrin knock-down). **d** Representative and **e** quantified β_3_ integrin western blot. **f** Cell surface α_v_β_3_ expression by flow cytometry. **g** [^3^H]ZMPZAT71 radiotracer binding assay. Results are representative or mean (± SD) of at least three independent experiments. Statistical analysis: *P < 0.05, **P < 0.01, ***P < 0.001.

In U87MG cells, we observed that total β_3_ integrin expression varied with cultivation time (Fig. 1a), more than doubling between 24 h and 72 h after cell seeding (Fig. 1a). Flow cytometry analysis confirmed that α_v_β_3_ integrin cell surface expression also increased with time (Fig. 1b). This trait was used initially to investigate the ability of [^3^H]ZMPZAT71 to differentiate between different α_v_β_3_ integrin expression levels. There was a clear time-dependent increase in specific radiotracer binding (Fig. 1c), reflecting the increased integrin expression (Fig. 1a, b).

α_v_β_3_ knock-down clones were developed to confirm that the radiotracer binds specifically to α_v_β_3_ integrin. Since β_3_ integrin forms heterodimers only with α_v_ or α_IIb_ [6], and cancer cells do not express α_IIb_, α_v_β_3_ knock-down clones can be generated by downregulating β_3_ expression. Screening of multiple clones identified clone 2F, which exhibited a 65 % reduction in β_3_ integrin (Fig. 1d, e) and a 44 % decrease in cell surface α_v_β_3_ integrin expression (Fig. 1f). Radiotracer binding decreased by 66 % in the 2F clones (Fig. 1g), in line with the flow cytometry and western blotting results (Fig. 1e, f). These data confirm that downregulation of α_v_β_3_ integrin produces a corresponding decrease in radiotracer binding. Overall, a good correlation (*r* ^2^ = 0.91) was observed between radiotracer binding and α_v_β_3_ integrin expression (Fig. S2).

### Radiotracer Binding Is Influenced by α_v_β_3_ Activation Status

**Fig. 2.**
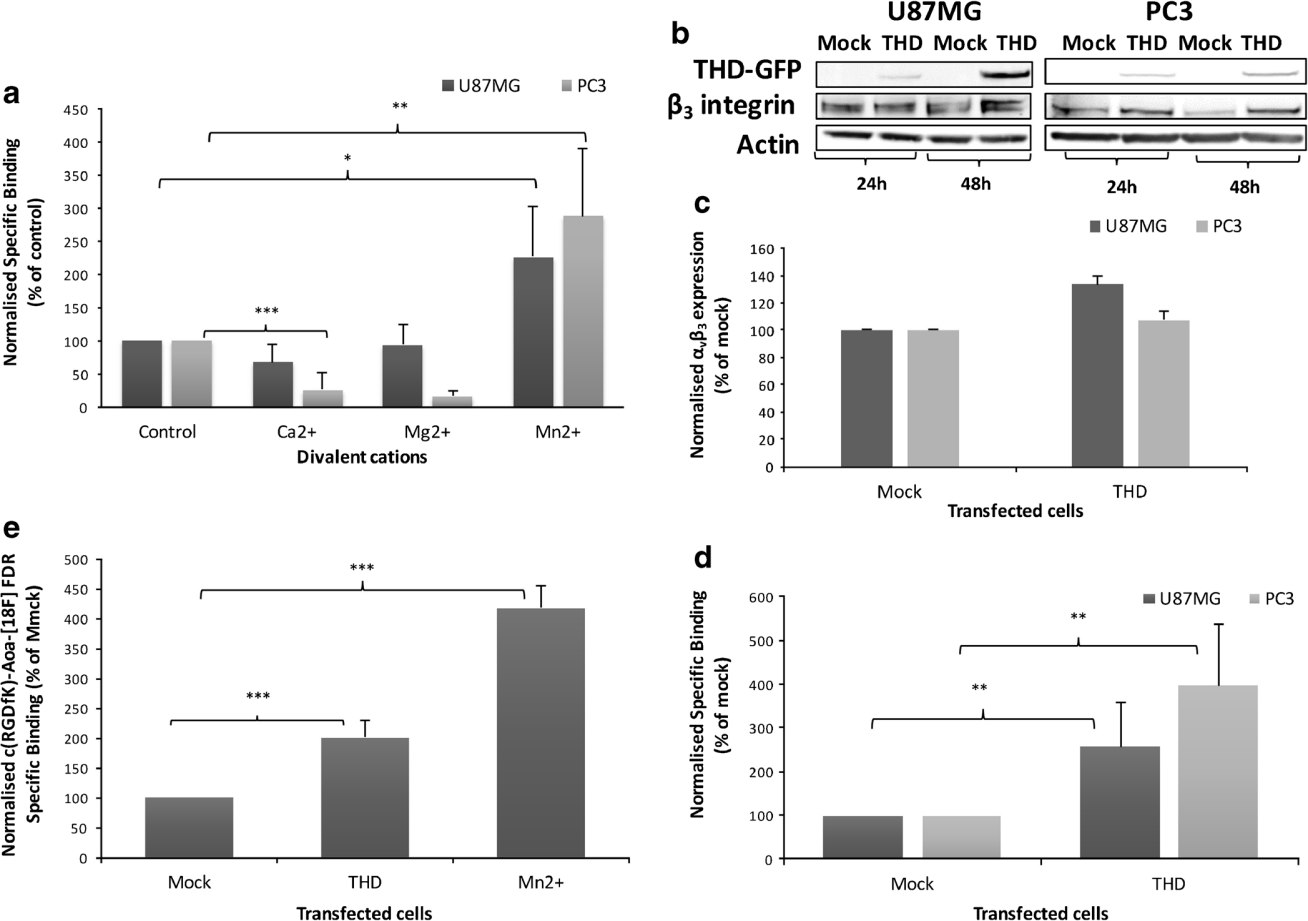
α_v_β_3_ integrin radiotracer binding is increased by receptor activation. **a** [^3^H]ZMPZAT71 radiotracer binding assay in the absence of metal ions (control) or in the presence of 1 mM Mn^2+^, Mg^2+^ or Ca^2+^. **b**–**e** Cells were either mock transfected or transfected with talin head domain (THD) linked to green fluorescent protein (GFP). **b** Western blot of β_3_ integrin and THD expression. **c** Cell surface expression of α_v_β_3_ by flow cytometry. **d** [^3^H]ZMPZAT71 and **e** [^18^F]FDR-Aoa-c(RGDfK) radiotracer binding assays. Results are representative or mean (± SD) of at least three independent experiments. Statistical analysis: *P < 0.05, **P < 0.01, ***P < 0.001.

It is well established that integrins are activated by Mn^2+^, minimally affected by Mg^2+^ and inhibited by Ca^2+^ [23]. Therefore, the effect of divalent cations on radiotracer binding was investigated to determine if the activation status of the receptor can influence [^3^H]ZMPZAT71 binding. Divalent cations were added to medium just prior to radiotracer, ensuring that cells were not exposed to these stimuli for longer than 30 min. Indeed, in the presence of Mn^2+^, radiotracer binding significantly increased in both PC3 and U87MG cell lines (Fig. 2a), despite there being no significant increase in cell surface α_v_β_3_ expression (data not shown). In contrast, Mg^2+^ did not increase radiotracer binding to either cell line, whereas Ca^2+^ decreased binding in both cell lines (Fig. 2a).

U87MG and PC3 cells were transfected with THD-GFP construct, a specific activator of integrin proteins [24], to determine whether the observed increase in radiotracer binding (Fig. 2a) is specifically due to integrin activation. Optimal transfection was observed after 24 h in PC3 cells and 48 h in U87MG cells (Fig. S3). THD expression had no significant effect on β_3_ integrin expression (Fig. 2b), although a small increase (33 and 8 %, respectively) in cell surface α_v_β_3_ expression was observed in U87MG and PC3 cells (Fig. 2c). THD transfection produced a significant increase in radiotracer binding in both cell lines; a 156 % increase was observed in U87MG and a 297 % increase in PC3 (Fig. 2d). These data are consistent with the activation effect observed with Mn^2+^. Together, these two datasets provide strong evidence that the activation status of α_v_β_3_ integrin has a significant influence on [^3^H]ZMPZAT71 binding. An equivalent experiment was therefore performed with [^18^F]FDR-Aoa-c(RGDfK) [21] to ascertain whether binding of RGD-based radiotracers is similarly influenced by integrin activation. Indeed, the results clearly show that THD transfection and Mn^2+^ both significantly increased specific binding (Fig. 2e) of this class of radiotracers. Correlation (*r* ^2^ = 0.83) was observed between radiotracer binding and α_v_β_3_ integrin activation status (Fig. S2).

### Increased Cell Surface Integrin Expression Does Not Necessarily Enhance α_v_β_3_ Integrin Radiotracer Binding

**Fig. 3.**
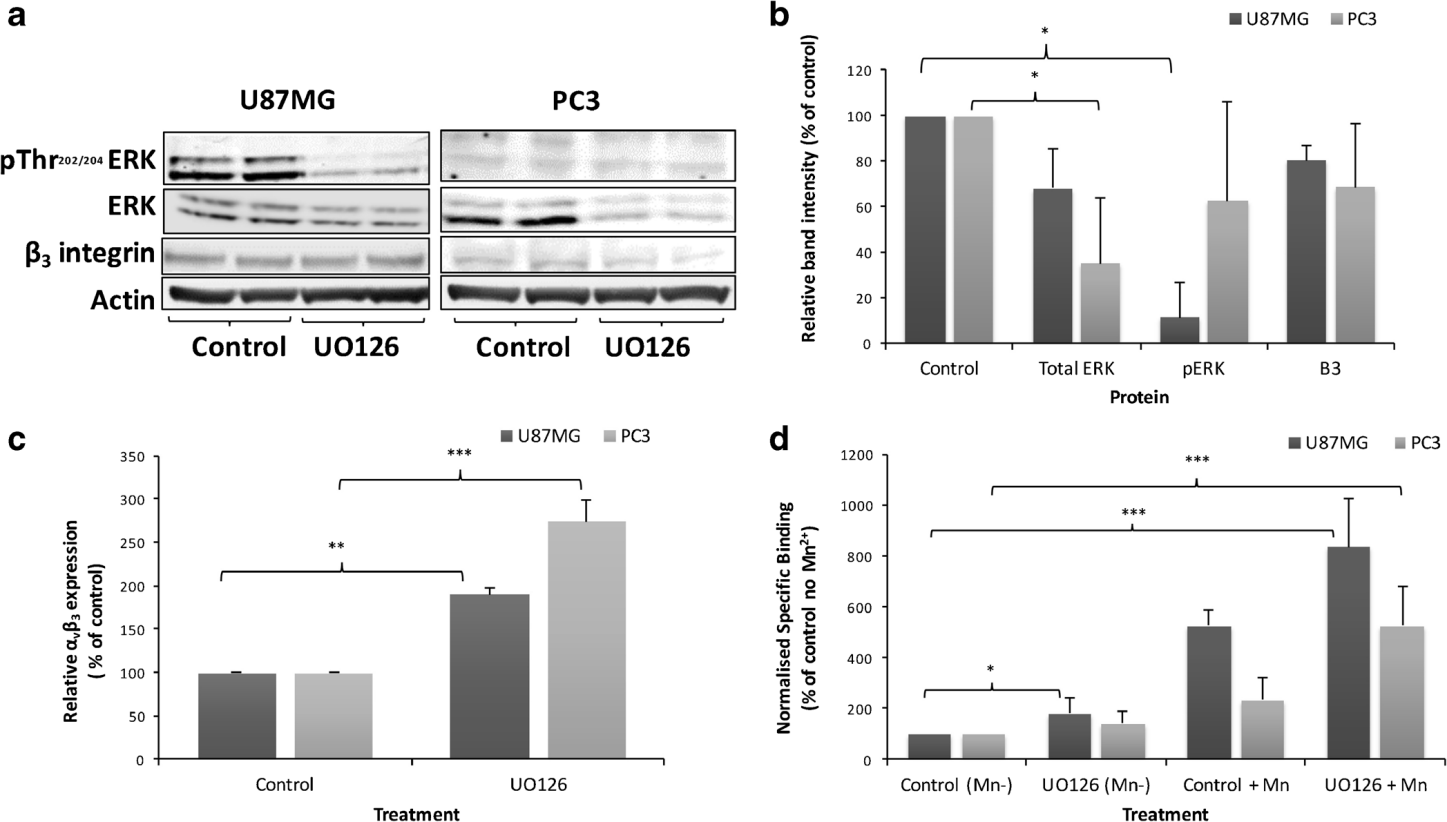
ERK inhibition increases cell surface expression of α_v_β_3_ but decreases its activation status. Cells were treated with 25 μM UO126 or vehicle for 24 h. **a** Representative and **b** quantitative western blots analysis of ERK, pERK and β_3_ integrin. **c** Cell surface α_v_β_3_ expression by flow cytometry. **d** [^3^H]ZMPZAT71 radiotracer binding assay performed in the presence (+) and absence (−) of Mn^2+^. Results are representative or mean (± SD) of at least three independent experiments. Statistical analysis: *P < 0.05, **P < 0.01, ***P < 0.001.

α_v_β_3_ integrin modulates signals from outside and inside cells. However, the impact of many signalling pathways on α_v_β_3_ activation is uncertain [25]. UO126, a potent and selective MEK1/2 inhibitor, was used to study the effect of this signalling pathway on α_v_β_3_ integrin. UO126 decreased ERK 1/2 phosphorylation by approximately 90 % in U87MG cells and resulted in both de-phosphorylation and degradation of ERK in PC3 cells (Fig. 3a, b). While UO126 had no significant effect on β_3_ expression (Fig. 3b), α_v_β_3_ cell surface expression significantly increased by 90 % in U87MG and 175 % in PC3 cells (Fig. 3c).

Radiotracer binding assays performed in the absence of Mn^2+^ detected an 82 % increase in binding in U87MG cells and a 42 % increase in PC3 cells after UO126 treatment (Fig. 3d). As expected (Fig. 2a), inclusion of Mn^2+^ increased radiotracer binding by 4.58- and 3.7-fold in UO126-treated U87MG and PC3 cells, respectively (Fig. 3d). These results demonstrate that an increase in cell surface α_v_β_3_ expression does not necessarily produce an equivalent increase in radiotracer binding, but this can occur if the integrin becomes activated. These data also indicate that α_v_β_3_ integrin radiotracers can report on the activation status of the receptor and that this is decreased by MEK1/2 inhibition.

### Modulation of α_v_β_3_ Integrin with Pharmacological Inhibitors

**Fig. 4.**
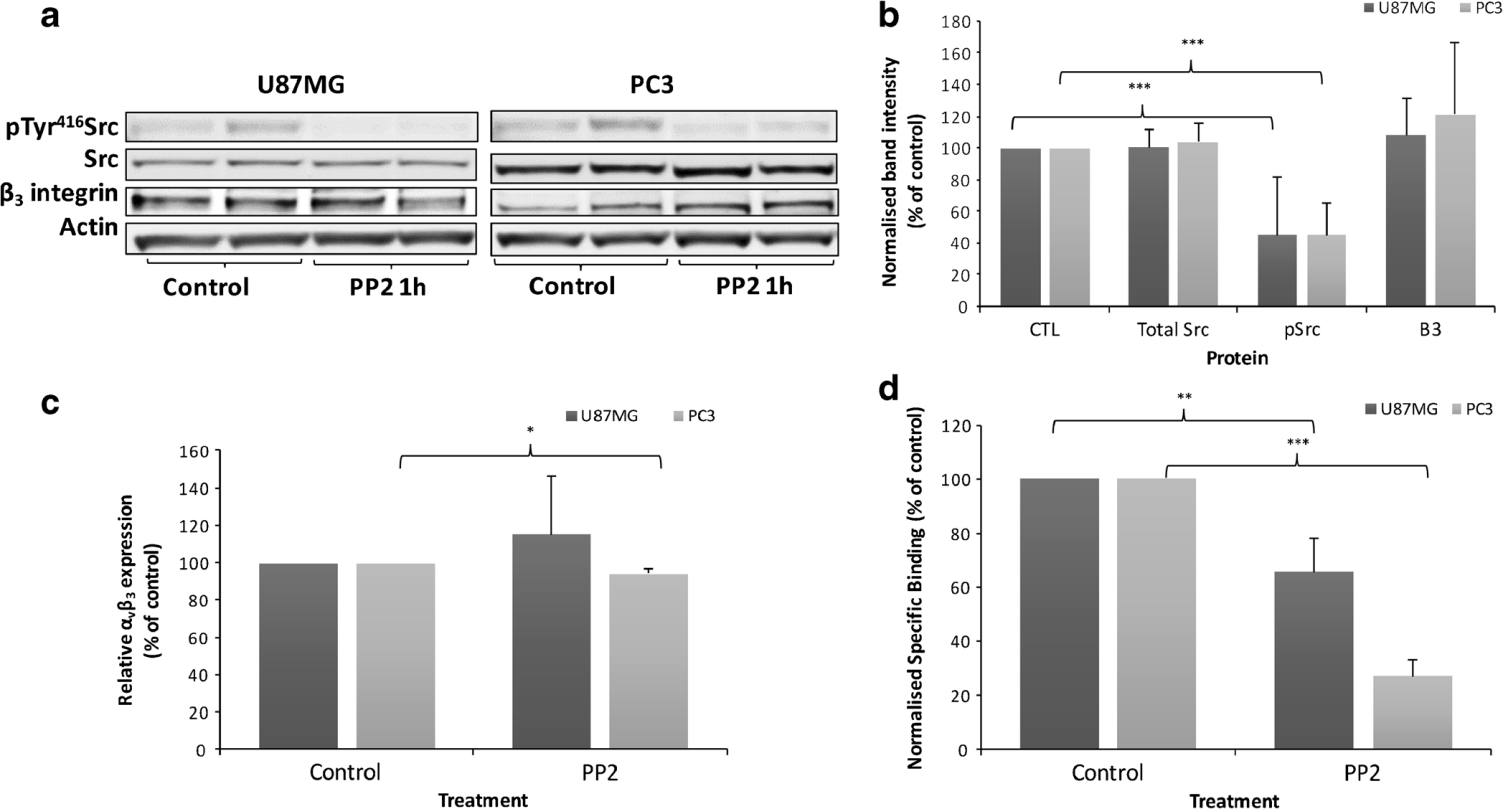
Src inhibition decreases α_v_β_3_ integrin radiotracer binding. Cells were treated with 10 μM PP2 or vehicle for 1 h. **a** Representative and **b** quantitative western blot analysis of Src, pSrc and β_3_ integrin. **c** Cell surface α_v_β_3_ expression by flow cytometry. **d** [^3^H]ZMPZAT71 radiotracer binding assay performed in the absence of Mn^2+^. Results are representative or mean (± SD) of at least three independent experiments. Statistical analysis: *P < 0.05, **P < 0.01, ***P < 0.001.

In light of the finding that α_v_β_3_ integrin radiotracers can convey information on the activation status of its target receptor, we investigated how select kinases involved in α_v_β_3_ signalling could modulate its activation status. Clustering of β_3_ integrins induces activation of Src *via* phosphorylation of Tyr^418^ [26], stimulating tumour growth and lymph node metastases [27]. The influence of Src was tested using PP2, a selective Src inhibitor (Fig. S4). PP2 induced a 45 % decrease in Src phosphorylation in both cell lines (Fig. 4a, b). Negligible changes were observed in β_3_ integrin levels (Fig. 4b) or cell surface α_v_β_3_ integrin expression (Fig. 4c). Radiotracer binding decreased by approximately 70 % in PC3 and 40 % in U87MG cells (Fig. 4d), suggesting that Src inhibition decreases α_v_β_3_ integrin activation. In contrast, inhibition of FAK phosphorylation using PF573228 had no significant effect on α_v_β_3_ integrin expression, localisation or radiotracer binding (Fig. S5).

**Fig. 5.**
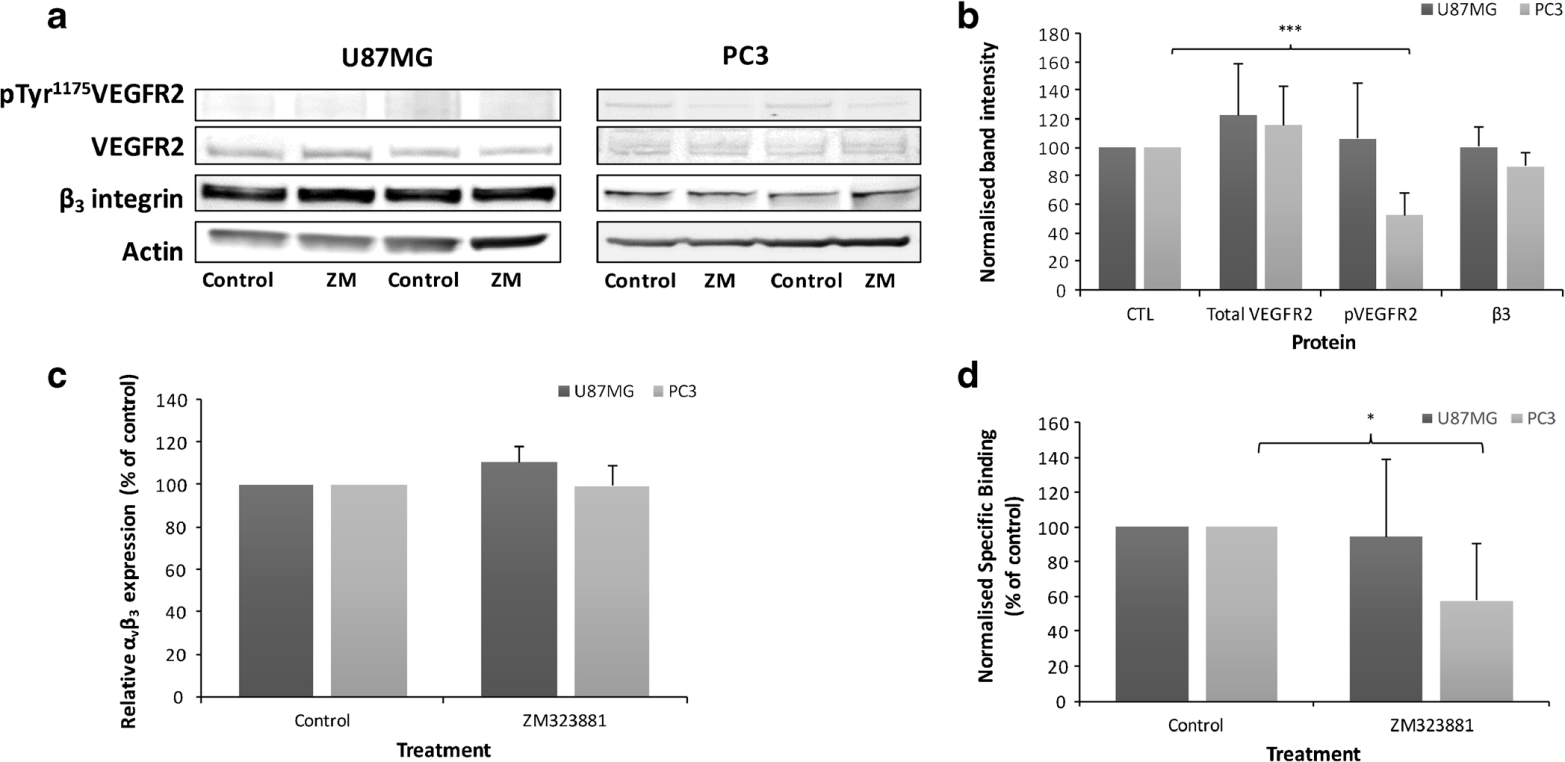
VEGFR2 inhibition decreases α_v_β_3_ integrin radiotracer binding. Cells were treated with 1 μM ZM323881 for 1 h or vehicle for 1 h. **a** Representative and **b** quantitative western blot analysis of VEGFR2, pVEGFR2 and β_3_ integrin. **c** Cell surface α_v_β_3_ expression by flow cytometry. **d** [^3^H]ZMPZAT71 radiotracer binding assay performed in the absence of Mn^2+^. Results are representative or mean (± SD) of at least three independent experiments. Statistical analysis: *P < 0.05, **P < 0.01, ***P < 0.001.

α_v_β_3_ integrin activation is dependent on VEGFR2 in endothelial cells [28]. ZM323881, a potent and selective VEGFR2 inhibitor, was used to investigate the role of this receptor on α_v_β_3_ integrin expression and activation. ZM323881 treatment decreased pTyr^1175^ VEGFR2 by 50 % in PC3 but had no significant effect in U87MG cells, likely due to the lower baseline VEGFR2 phosphorylation level detected. No significant changes in β_3_ integrin expression (Fig. 5a, b) or α_v_β_3_ integrin expression (Fig. 5c) were observed in either cell line. A 40 % decrease in radiotracer binding was observed in PC3 cells, but no significant change was detected in U87MG cells (Fig. 5d). Hence, downregulation of VEGFR2 signalling is clearly linked to decreased α_v_β_3_ integrin activation in cells. Importantly, inclusion of Mn^2+^ after cells had been treated with VEGFR2 (or Src) inhibitors significantly increased radiotracer binding (data not shown). This demonstrates that the receptor still retains the capacity to be activated and that the decreased radiotracer binding after drug treatment is due to reduced receptor activation status.

The understanding that α_v_β_3_ integrin radiotracer binding is influenced by both receptor expression and activation is an important advance. Table 1 summarises the effect of each treatment used herein on integrin expression and activation. Expressing radiotracer binding as a function of cell surface α_v_β_3_ integrin expression produces a new metric that permits a direct comparison of the effect of diverse intracellular and extracellular treatments on α_v_β_3_ integrin activation status.

**Table 1:** Expressing α_v_β_3_ integrin radiotracer binding as a function of cell surface expression produces a measure of integrin activation

	**Relative α**_**v**_ **β**_**3**_ **expression**		**Relative radiotracer binding**		**Activation ratio (binding/expression)**		**Effect on α**_**v**_ **β**_**3**_ **activity**
**Cell line**	**PC3**	**U87MG**	**PC3**	**U87MG**	**PC3**	**U87MG**	**−**
**Treatment**							
**Control**	100	100	100	100	1.00	1.00	−
**Mn** ^**2+**^	100	100	288	227	**2.88**	**2.27**	**Activation**
**THD**	108	133	397	256	**3.68**	**1.92**	**Activation**
**UO126**	274	190	142	182	*0.52*	*0.95*	*Inhibition*
**UO126+Mn** ^**2+**^	274	190	526	834	**1.92**	**4.39**	**Activation**
**PP2**	92	115	27	66	*0.29*	*0.57*	*Inhibition*
**PF573228**	102	114	124	102	1.22	0.89	No apparent change
**ZM323881**	99	110	58	95	*0.59*	*0.86*	*Inhibition in PC3 cells*

Bold text has been used to represent increased radiotracer binding and italic text to highlight decreased radiotracer binding

Normalised α_v_β_3_ integrin radiotracer binding after each treatment was expressed as a function of the normalised cell surface α_v_β_3_ integrin expression. The resulting ratio provides a measure of α_v_β_3_ integrin activation compared to control cells. A ratio >1 signifies activation, whereas a ratio <1 represents inhibition.

## Discussion

There is an urgent need for sensitive, accurate and robust imaging methods to report on tumour angiogenesis since the current success rate of anti-angiogenic therapies varies considerably between both tumour types and individual patients. α_v_β_3_ integrin is well recognised as an appropriate target for providing pertinent molecular imaging information, as the receptor is highly expressed on angiogenic endothelial cells and a validated target for assessing tumour angiogenesis [10]. Correlation between baseline α_v_β_3_ integrin expression and binding of α_v_β_3_ integrin-targeted radiotracers has been established in multiple preclinical and clinical studies [11–15], demonstrating their suitability to detect tumours with angiogenic potential. On the other hand, while multiple preclinical studies have utilised α_v_β_3_ integrin radiotracers to assess response to anti-angiogenic therapies [17, 18, 29–32], most have either consciously not reported correlation between radiotracer binding and receptor expression [29, 30, 32] or have reported a lack of correlation [17, 18]. This strongly suggests that there are uncharacterised factor(s) that can influence radiotracer uptake in cells/tumours. In this study, we directly demonstrate for the first time that radiotracer binding can be attributed to both α_v_β_3_ integrin activation status and expression, providing a rationale for the documented discrepancy between radiotracer binding and receptor expression after therapy.

Several lines of evidence demonstrate that [^3^H]ZMPZAT71 binds specifically to α_v_β_3_ in cells and the magnitude of binding reflects receptor expression. Firstly, in U87MG cells, [^3^H]ZMPZAT71 binding increased in a temporal manner in parallel with α_v_β_3_ integrin (Fig. 1). Secondly, in β_3_ integrin knock-down cells, proportionate decreases in radiotracer binding and receptor knock-down were observed (Fig. 1). Finally, UO126 treatment produced increases in both cell surface α_v_β_3_ integrin expression and radiotracer binding when Mn^2+^ was present (Fig. 3). These results are in agreement with previous studies showing a direct relationship between α_v_β_3_ integrin expression and a α_v_β_3_-targeted radiotracer uptake in xenograft [14, 15] and clinical studies [11–13]. Taken together, these data provide strong evidence of a direct relationship between baseline receptor expression and binding of multiple distinct α_v_β_3_ integrin radiotracers.

Significant increases in α_v_β_3_ integrin radiotracer binding were observed in both cell line models after integrins were activated *via* two independent mechanisms (Fig. 2). Mn^2+^ binds to metal ion-dependent adhesion site (MIDAS) on the β subunit extracellular domain [7], producing a conformation change that increases α_v_β_3_ affinity/avidity for ligands [33]. The opposite effect was observed with Ca^2+^, confirming that integrin activity can also be decreased. Talin head domain selectively activates integrins by binding to their intracellular domain and changing their conformation [34]. The data presented herein indicate that radiotracer binding is sensitive to integrin activation induced either by extracellular or intracellular activation. Indeed, this is apparently a class effect since binding of two distinct α_v_β_3_ integrin binding radiotracers, [^3^H]ZMPZAT71 and [^18^F]FDR-Aoa-(RGDfK), was enhanced. Taken together, these two datasets provide very strong evidence that radiotracer binding is sensitive to integrin activation status in cells and that these radiotracers can report on α_v_β_3_ integrin activation status. To our knowledge, this is the first study to directly demonstrate this.

Two xenograft studies have also reported changes in binding of α_v_β_3_ integrin radiotracers that cannot be accounted for by altered α_v_β_3_ integrin expression [17, 18]. The most likely interpretation of the study data is that α_v_β_3_ integrin activation is also modulated *in vivo*. In the first study, an U87MG xenograft was treated with the Src inhibitor dasatinib. [^64^Cu]-DOTA-c(RGDfK) radiotracer uptake was decreased in the absence of any effect on total or cell surface α_v_β_3_ integrin expression [18]. This result can be accounted for by the decrease in α_v_β_3_ integrin activation caused by Src inhibitors (Fig. 4d and Table 1). The second study involved an A431 xenograft treated with bevacizumab. Increased [^68^Ga]-NODAGA-c(RGDfK) binding was observed despite a 50 % decrease in α_v_β_3_ integrin expression [17]. The authors attributed the increased binding to integrin activation and/or enhanced tumour perfusion. On the other hand, an MDA-MB-435 xenograft study showed that [^18^F]FPPRGD2 binding and α_v_β_3_ integrin staining both decreased after treatment with ZD4190, a potent inhibitor of VEGFR1 and VEGFR2 inhibitor [31], in agreement with the observed correlation between baseline integrin expression and radiotracer binding (Fig. 1) [11, 12, 14, 15]. Other *in vivo* studies have not assessed α_v_β_3_ integrin expression [29, 30, 32] but have demonstrated decreased radiotracer binding after treatment with VEGFR2 inhibitors. In light of the data presented in Fig. 5, it seems likely that the observed decrease in radiotracer binding could be attributed to decreased α_v_β_3_ integrin activation.

Prior to this study, the effect of individual cell signalling proteins on α_v_β_3_ integrin activation was not clear [4, 25]. Our finding that α_v_β_3_ integrin binding radiotracers can report on α_v_β_3_ integrin activation status allows this otherwise inaccessible information to be directly revealed. The results clearly demonstrate that inhibition of MEK1/2, Src and VEGFR2, but not FAK, decreases α_v_β_3_ integrin activation (Table 1). The effect of the Src inhibitor is in line with a U87MG xenograft study that intimates that the Src inhibitor dasatinib may decrease α_v_β_3_ integrin activation [18]. It is well established that Src family kinases are activated by integrins through the β_3_ cytoplasmic tail. The data presented herein demonstrate that Src also plays a role in integrin activation, although it is not clear if this is a direct or indirect effect. The inhibitory effect of VEGFR2 inhibitors shown here (Fig. 5) provides a molecular rationale for several xenograft studies that have shown reduced radiotracer uptake [30–32] after VEGFR2 inhibitor treatment. VEGFR2-α_v_β_3_ integrin association is important for full VEGFR2 activity and subsequent activation of intracellular signalling pathways [2]. As VEGFR2 autophosphorylation is crucial for VEGFR2-α_v_β_3_ interaction, VEGFR2 inhibitors can reduce α_v_β_3_ integrin activation. The magnitude of this effect appears to be dependent on the basal level of VEGFR2 phosphorylation, since an inhibitory effect was not observed in U87MG cells, which contain lower levels of VEGFR2 phosphorylation. Increased cell surface expression of α_v_β_3_ and other integrin subunits following UO126 treatment have been reported previously [35, 36]. However, its inhibitory effect on α_v_β_3_ integrin activation has not been documented. One possibility is that the increased cell surface expression of α_v_β_3_ integrin is a direct consequence of the decreased integrin activity.

The data reported herein have significant implications for assessing response to anti-angiogenic therapies in clinical studies. Studies need to consider both the expression level and activation status of integrins. This will make data interpretation more challenging but also raises new possibilities. Challenges include the confounding factors arising from combining anti-angiogenics with standard chemotherapies, which could either alter the expression level or activation status of α_v_β_3_ integrin. Opportunities include the possibility of studying binding of α_v_β_3_ integrin radiotracers to either the tumour vasculature or tumour cells that express α_v_β_3_ integrin, to assess the efficacy of Src, VEGFR2 and MEK inhibitors. This study provides valuable insight into the molecular mechanism of modulation of integrin signalling pathways in response to anti-cancer agents, but further work is required to take full advantage of these new opportunities to use α_v_β_3_ integrin-targeted radiotracers for assessing response to therapy.

## Conclusion

Binding of radiotracers to cells corresponds with both changes in α_v_β_3_ integrin expression and activation status. These results are applicable to both RGD-labelled peptides and small molecule antagonists of α_v_β_3_ integrin, indicating that this is a general effect, pertinent to this entire class of radiotracers. These findings provide a rationale for the documented discrepancy between radiotracer binding and receptor expression after therapy in several xenograft studies and have important consequences for imaging α_v_β_3_ integrin *in vivo*. Some tumours that express this receptor may not have significant uptake of these radiotracers if the receptors are not activated. Moreover, these radiotracers appear to have significant potential to assess response to treatment with a range of classes of anti-cancer drugs (*e.g.,* ERK, Src and VEGFR2 inhibitors) by providing early guidance on the efficacy of these agents in clinical studies.

## Electronic supplementary material

ESM 1 (PDF 534 kb)

## Abbreviations

cRGDfK: Cyclo(Arg-Gly-Asp-d-Phe-Lys) peptide
ERK: Extracellular signal regulated kinase
GFP: Green fluorescent protein
FAK: Focal adhesion kinase
MEK: Mitogen-activated protein kinase kinase
MIDAS: Metal ion-dependent adhesion site
PC: Prostate cancer
PET: Positron emission tomography
THD: Talin head domain
VEGF: Vascular endothelial factor
VEGFR2: Vascular endothelial factor receptor 2
