# Supplementary material for: Binding of αvβ3 Integrin-Specific Radiotracers Is Modulated by Both Integrin Expression Level and Activation Status
Source: Mol Imaging Biol. 2017 Jul 10;20(1):27–36. doi: 10.1007/s11307-017-1100-z (PMC5775384; doi:10.1007/s11307-017-1100-z)
Supplement: Supplementary file 1 — (PDF 534 kb) [file 11307_2017_1100_MOESM1_ESM.pdf]

Supplementary data – 21st March 2017

Binding of $\alpha_v\beta_3$ Integrin Specific Radiotracers is Modulated by Both Integrin Expression Level and Activation Status

Journal: Molecular Imaging and Biology

Alexandra Andriu[1], Julie Crockett[2], Sergio Dall'Angelo[3], Monica Piras[3], Matteo Zanda[3], Ian N. Fleming[1,4]

[1] Aberdeen Biomedical Imaging Centre, Institute of Medical Sciences, University of Aberdeen, Aberdeen AB25 2ZD, UK

[2] Arthritis and Musculoskeletal Medicine Research Programme, Division of Applied Medicine, Institute of Medical Sciences, University of Aberdeen, Aberdeen, AB25 2ZD UK

[3] Kosterlitz Centre for Therapeutics, Institute of Medical Sciences, University of Aberdeen, Aberdeen, AB25 2ZD, Scotland, UK

[4] Correspondence to Ian N. Fleming, Institute of Medical Sciences, Aberdeen AB25 2ZD, UK; Email: i.n.fleming@abdn.ac.uk Tel # (+44) (0)1224438357, orchid number 0000-0001-9505-6418

Methods

Cell cytotoxic assays

Cells were seeded at 5,000/well in 96 well plates and left to attach overnight. Cytotoxicity assays were performed essentially as described, in triplicate wells [1]. Stock solutions of UO126, PP2, PF573228 and ZM323881 were prepared in DMSO. A range of concentrations of PP2 and ZM323881 was added to the cells and left for 72 h. The cytotoxic effect of UO126 and PF228 was confirmed using established IC_{50} concentrations. Cell viability was evaluated by incubation in RPMI containing 10 % MTT (3-(4,5 dimethylthiazol-2-yl)-2,5-diphenyl tetrazolium bromide). After 2-4 h incubation medium was aspirated. The formazan product was solubilized in DMSO and detected at A560 using a microplate reader. Inhibitory concentrations 50 (IC_{50}) were calculated using Graph PAD Prism v.5 software.

Results

Cell cytotoxic assays

The efficacy of the various treatments used in this study were initially assessed using cell cytotoxicity assays. The IC_{50} value for each inhibitor is presented in **Table 1**. The IC_{50} values were used to guide mechanistic studies to assess the effect of each agent on its target protein (e.g. Src phosphorylation).

Inhibitor (concentration)	Target protein	Cell line	
		**U87MG**	**PC3**
**UO126 (µM)**	ERK1/2	5	10
**PP2 (µM)**	Src	20	12
**PF573228 (nM)**	FAK	30-100	30-100
**ZM323881 (µM)**	VEGFR2	17	88

Table 1. IC_{50} for UO126, PP2, PF228 and ZM323881 in U87MG and PC3 cell lines. *IC_{50} for UO126 and PF228 were taken from published literature [2] and [3].

Determining total, specific and non-specific binding of $\alpha_v\beta_3$ integrin radiotracers

Radiotracer binding was measured in the presence and absence of 10µM non-radiolabelled cRGDfK peptide (**Fig. S1**). Binding in the absence of cRGDfK represents total binding. cRDGfK binds to $\alpha_v\beta_3$ integrin with high affinity, and 10µM was included so that it is present in a large excess compared to the radiotracer under investigation. Radiotracer binding in the presence of cRGDfK therefore

represents background (non-specific) binding. Specific binding is the difference between total binding and background binding.

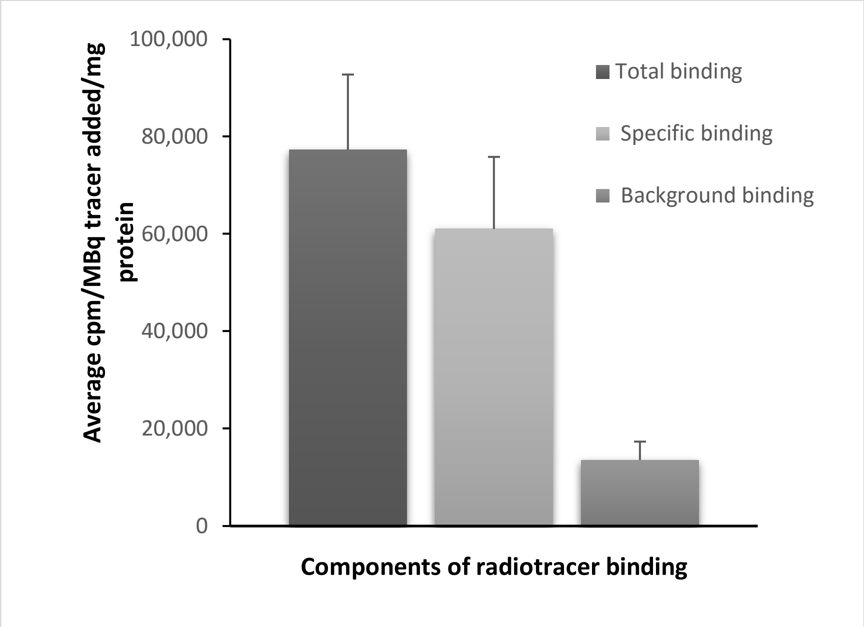

Fig. S1. Calculating total, specific and non-specific binding of $\alpha_v\beta_3$ integrin radiotracers. Fig S1 demonstrates how specific binding was calculated for the 48 h time point in Fig. 1c of the manuscript. Specific binding is calculated by subtracting non-specific binding (binding in presence of cRGDfK) from total binding (binding in absence of cRGDfK).

Correlation between $\alpha_v\beta_3$ integrin radiotracer binding and either $\alpha_v\beta_3$ integrin expression or activation status

The relationship between $\alpha_v\beta_3$ integrin radiotracer binding and $\alpha_v\beta_3$ integrin expression was established using data from Fig 1 and Fig 3. For each datapoint the relative radiotracer binding was plotted against $\alpha_v\beta_3$ integrin expression (Fig S2). Similarly, the relationship between $\alpha_v\beta_3$ integrin radiotracer binding and $\alpha_v\beta_3$ integrin activation status (Fig S2) was investigated by plotting $\alpha_v\beta_3$ integrin radiotracer binding values from Fig 2 against the established effect of each metal ion on $\alpha_v\beta_3$ integrin activation, as measured by binding of purified receptor to vitronectin [4]. Correlations were observed in both graphs. The impact of these correlation graphs is, however, restricted by the limited number of independent datapoints in each plot.

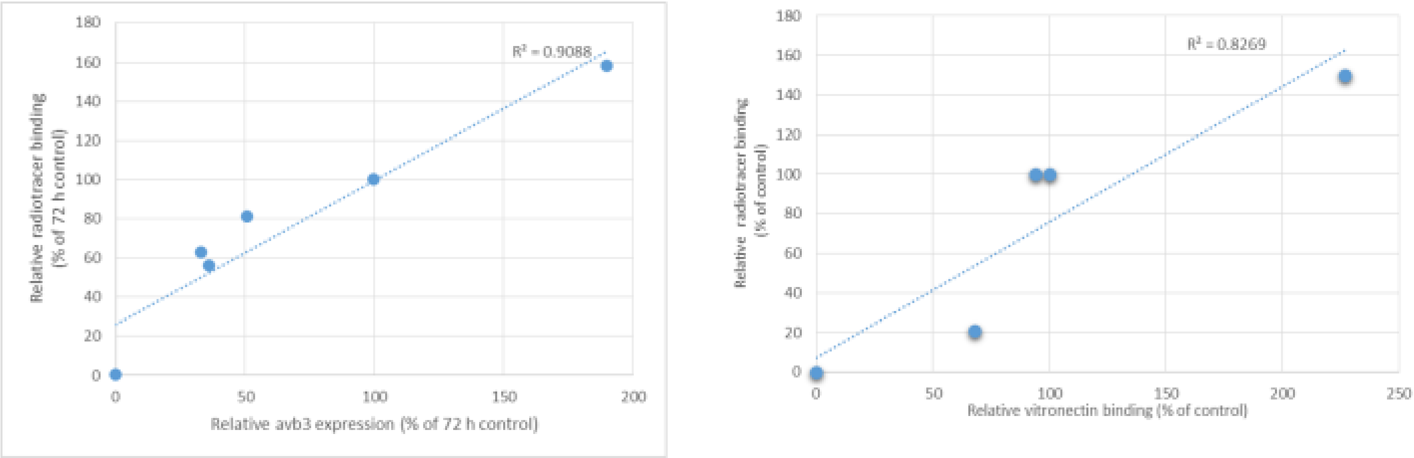

Fig. S2. Relationship between radiotracer binding and either $\alpha_v\beta_3$ integrin expression or activation status

In the left hand panel datapoints generated from U87MG cells in Fig 1 and Fig 3 were plotted. For each datapoint radiotracer binding was plotted against $\alpha_v\beta_3$ integrin expression as assessed by flow cytometry. All of these radiotracer binding assays were performed in the presence of 1mM Mn^{2+} to ensure that $\alpha_v\beta_3$ integrin activation status had no influence on radiotracer binding. In the right hand panel datapoints from U87MG cells in Fig 2 were plotted. Radiotracer binding was assessed after treatment with different metal ions. For each datapoint radiotracer binding was plotted against binding of purified receptor to vitronectin, a well-established measure of integrin binding affinity.

Determining THD transfection efficiency

THD-GFP transfected cells were analysed visually under a microscope. For each data point the total number of cells (transfected or non-transfected) and the number of transfected cells (green) counted in several fields of view (minimum of 500 cells counted). These numbers were used to calculate the

percentage of transfected cells at times ranging from 4 – 48 h (**Fig. S3**). Based on these values, an optimal transfection time was chosen for each cell line (24 h for PC3 and 48 h for U87MG cells). Western blot analysis confirmed strong expression of THD-GFP (see Fig. 2b of manuscript) at the predicted molecular weight of the THD-GFP construct.

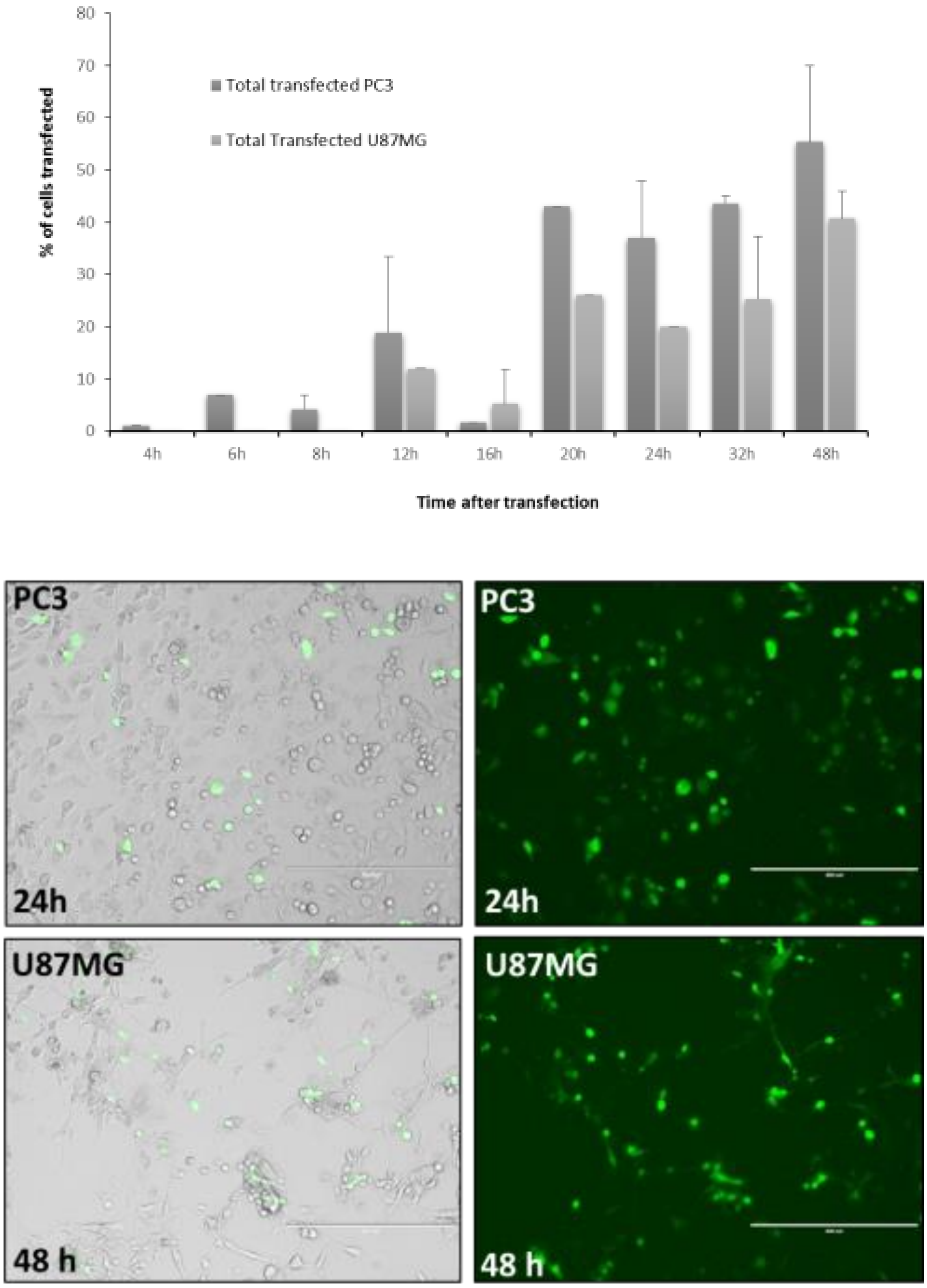

Fig.S3: Optimising GFP-THD cell transfection time. THD transfection efficiency was determined by calculating the percentage of green (GFP-THD transfected) cells. Upper panel depicts average transfection efficiency ± s.d. from 3 independent experiments. The lower panels show an example

of a typical field of view of transfected PC3 and U87MG cells. The left hand image represents a transmitted light field of view (to determine the total number of cells) of typical transfected cell populations. The right hand image represents a fluorescent image (to determine the number of transfected cells) of the same cell population.

Optimising use of PP2 on Src phosphorylation

A range of drug concentrations were tested to determine the maximal effect of drug inhibition of each target of interest (pERK, pFAK, pSrc and pVEGFR2). For example PP2 treatment was used at concentrations ranging from 1 nM to 10 µM (**Fig. S4**). Cells started to detach from the plates when PP2 concentration higher than 10 µM were employed, and there was also a decrease in total Src expression. Therefore the dose that reduced pTyr^{416} Src with minimal effect on total Src expression or cell detachment (i.e.10 µM) was selected for further experiments.

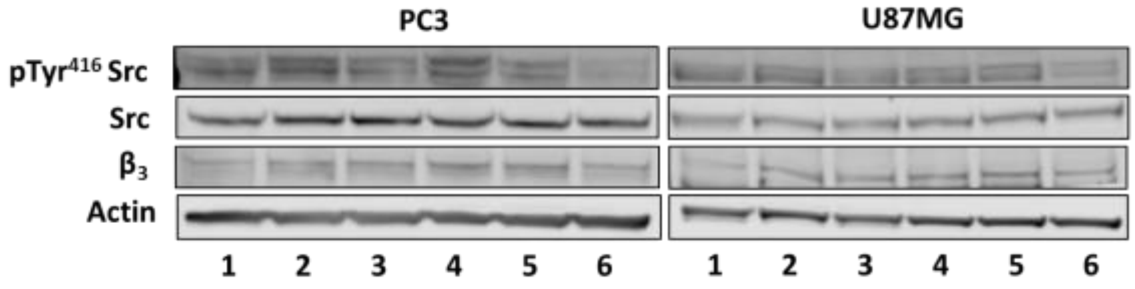

Fig. S4: Optimising the use of PP2 to inhibit Src phosphorylation. Effect of 1 h PP2 treatment on pTyr^{416} Src, total Src, β_3 integrin in PC3 and U87MG cells. Lane 1 = control, lane 2 = 1nM PP2, lane 3 = 10nM PP2, lane 4 = 100nM PP2, lane 5 = 1 µM PP2, lane 6 = 10 µM PP2. Result is representative of three independent experiments.

Influence of FAK inhibition on $\alpha_v\beta_3$ integrin

Following integrin activation and clustering, Focal Adhesion Kinase (FAK) associates with and is phosphorylated by the cytoplasmic domain of integrins promoting cell spreading and migration. The effect of FAK inhibition was investigated by using PF573228 (PF228), a potent and selective FAK inhibitor. PF228 decreased pTyr^{397} FAK by approximately 50 and 70 % in U87MG and PC3 cells, respectively (**Fig. S5a** and **Fig. S5b**). No significant effects were observed on β_3 integrin expression

F**ig. S5b**), $\alpha_v\beta_3$ integrin expression (**Fig. S5c**) or radiotracer binding (**Fig. S5d**) were observed, indicating that FAK inhibition has little effect on $\alpha_v\beta_3$ integrin activation.

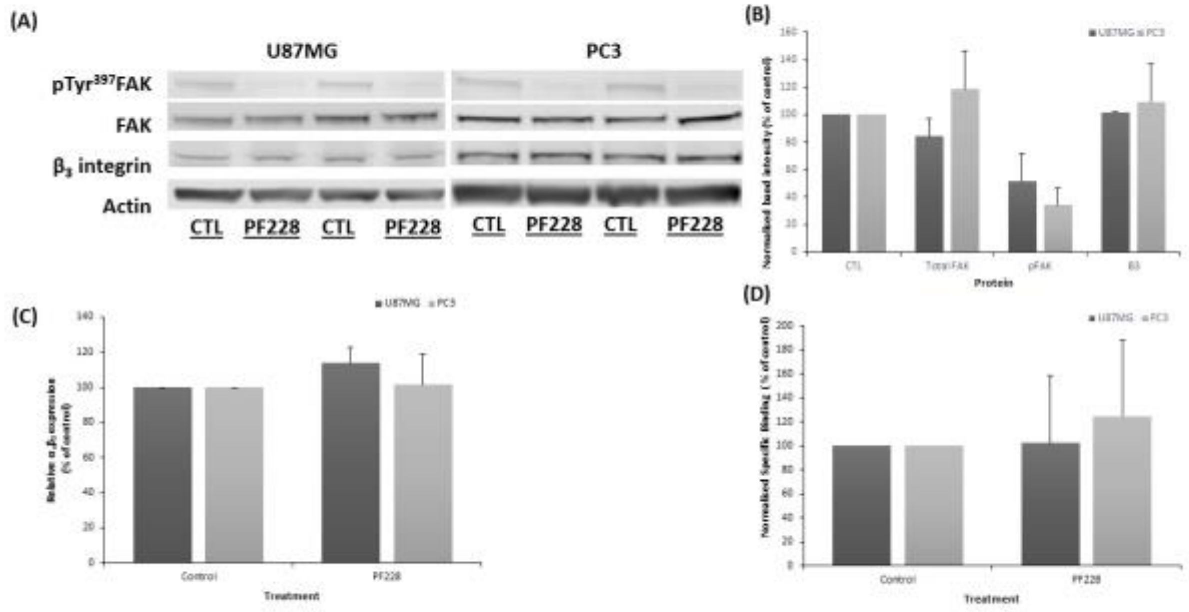

Fig. S5: FAK inhibition has no significant effect on $\alpha_v\beta_3$ integrin radiotracer binding

Cells were treated with 500nM PF228 or vehicle for 1 h. (a) Representative and (b) quantitative western blots analysis of FAK, pFAK and β_3 integrin. (c) Cell surface $\alpha_v\beta_3$ expression by flow cytometry. (d) [^{3}H]-ZMPZAT71 radiotracer binding assay performed in the absence of Mn^{2+}. Results are representative or mean (+/- SD) of at least three independent experiments.

Supplementary References

1. Lamidi OF, Sani M, Lazzari P, Zanda M, Fleming IN. The tubulysin analogue KEMTUB10 induces apoptosis in breast cancer cells via p53, Bim and Bcl-2. J Cancer Res Clin Oncol 2015;141:1575-83.

2. Woods D, Cherwinski H, Venetsanakos E, Bhat A, Gysin S, Humbert M, Bray PF, Saylor VL, McMahon M. Induction of ß3-Integrin Gene Expression by Sustained Activation of the Ras-Regulated Raf–MEK–Extracellular Signal-Regulated Kinase Signaling Pathway. Molecular and Cellular Biology 2001;21:3192-205.

3. Slack-Davis J, Martin KH, Tilghman RW, Iwanicki M, Ung EJ, Autry C, Luzzio MJ, Cooper B, Kath JC, Roberts WG, Parsons JT. Cellular characterization of a novel focal adhesion kinase inhibitor. J Biol Chem 2007;282; 14845-52

4. Legler DF*, Wiedle G, Ross FP, Imhof BA. Superactivation of integrin $\alpha_v\beta_3$ by low antagonist concentrations J Cell Sci 2001; 114;1545-1553
